# CMV-Related Gastric Ulcer and Gastroduodenitis in an Immunocompetent Patient: A Case Report and Literature Review

**DOI:** 10.1155/2021/3513223

**Published:** 2021-11-12

**Authors:** Andrawus Beany, Tova Rainis

**Affiliations:** ^1^Division of Gastroenterology, Bnai Zion Medical Center, Haifa, Israel; ^2^The Ruth and Bruce Rappaport Faculty of Medicine, Technion, Haifa, Israel

## Abstract

**Background:**

Cytomegalovirus (CMV)- related gastroduodenal infection is rare in immunocompetent hosts, and although it is considered a self-limiting condition in most cases, there is scarce literature to assert its management. *Case Presentation*. We report a case of a 66-year-old immunocompetent male patient diagnosed with a giant gastric ulcer caused by CMV infection. The ulcer manifested as refractory vomiting and melena. Rapid and full resolution was observed on proton-pump inhibitor (PPI) monotherapy.

**Conclusion:**

Gastric CMV infection might mimic an advanced gastric tumor in individuals with an intact immune system. The condition is rare, and the diagnosis is challenging and oftentimes overlooked. However, a rapid resolution has been documented in all cases, even without antiviral therapy.

## 1. Background

Cytomegalovirus (CMV), a DNA virus and a member of the Herpesviridae family, is related to one of the most prevalent infectious diseases. The seroprevalence rates, as an evidence of prior infection with CMV, range between 40 to 100 percent of the adult population [1] and increase with age [2].

The spectrum of diseases caused by the virus in humans is diverse, and most cases are asymptomatic or experience an indolent course with nonspecific symptoms [3]. Acute CMV infection may manifest as infectious mononucleosis, or, less commonly, as a systemic disease with significant morbidity [4]. Single organ involvement; colitis, encephalitis, and myocarditis among others; or multisystemic disorders have been reported [5], mainly in patients with immunosuppression. Immunocompetent hosts, however, usually have a mild and self-limiting disease. Viral reactivation is common among critically ill immunocompetent patients [6] and immunocompromised individuals [7] and is associated with severe morbidity and high mortality rate.

In this paper, we report a case of a rare manifestation of gastrointestinal CMV infection in an immunocompetent patient, followed by a review of the literature.

## 2. Case Presentation

A 66-year-old male with a 3-week history of nausea and vomiting was admitted. He also reported a one-month history of weakness, night sweats, anorexia, and diarrhea.

Lab work performed prior to admission was notable for CMV-specific IgM and IgG antibodies and an elevated C-reactive protein (CRP). A left lower lobe consolidation was demonstrated in a chest X-ray. He was started on oral cefuroxime and roxithromycin for a diagnosis of lobar pneumonia. However, he continued to suffer from nausea and vomiting.

His past medical history included hypertension, treated with angiotensin converting enzyme inhibitor (ACE-I) and hydrochlorothiazide. The patient denied personal history of smoking, alcohol abuse, and the use of aspirin or nonsteroidal anti-inflammatory drugs (NSAIDs).

His vital signs were normal, and physical exam on admission was only notable for scant petechial skin eruption.

In-hospital complete blood count (CBC) and chemistry were only notable for mild leukocytosis, without reactive lymphocytosis, and a slightly elevated CRP.

Further infectious workup was performed, including blood and urine cultures, serology for *Brucella*, hepatitis B and C viruses, human-immunodeficiency virus, *Coxiella burnetii*, and leptospira, and stool *Clostridium difficile* toxin assay. Results returned negative in due course; however, the polymerase chain reaction (PCR) for CMV-DNA in the serum was positive (1,278 IU/ml).

During the second day of admission, the patient reported tarry stools and a CBC showed a 2 gr/dl decrease in hemoglobin.

**Figure 1:**
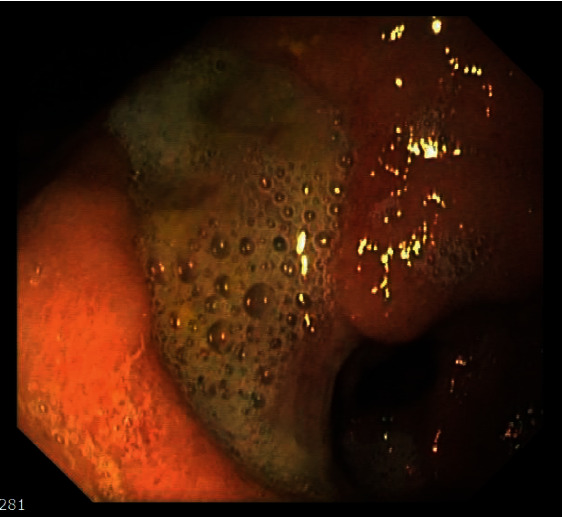
Endoscopy showing the gastric ulcer.

An esophagogastroduodenoscopy (EGD) revealed a large, irregularly shaped, deep ulcer in the gastric antrum, involving the angular incisure and the prepyloric region, with a deformation and narrowing of the pylorus (Figure 1). A few adjacent erosions were also seen. Biopsies were obtained from the stomach and duodenum. Colonoscopy was only notable for diverticulosis.

**Figure 2:**
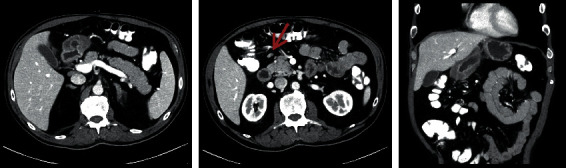
Computed tomography showing antral-wall thickening and mesenteric lymphadenopathy (arrow).

Abdominal computed tomography scans revealed diffuse antral-wall thickening with mild mesenteric lymphadenopathy (Figure 2).

**Figure 3:**
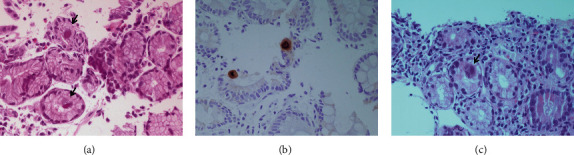
The patient's histological findings. (a) H&E staining showing chronic gastritis and scattered large cells with intranuclear and intracytoplasmatic inclusion bodies (arrows); (b) positive IHC in a representative duodenal specimen; (c) H&E staining showing duodenitis and large cells with inclusion bodies (arrow).

Staining of the gastric biopsy specimens with hematoxylin and eosin (H&E) revealed severe chronic active gastritis with ulceration and scattered large cells with inclusion bodies, characteristic of CMV infection (Figure 3(a)). Immunohistochemistry (IHC) using monoclonal CMV antibodies was also positive (Figure 3(b)). Staining for *Helicobacter pylori* bacilli was negative. Duodenal mucosa staining yielded duodenitis and scattered large cells with inclusion bodies (Figure 3(c)).

The patient was commenced on oral esomeprazole 40 mg. On this regimen, his gastrointestinal (GI) and systemic symptoms rapidly and fully resolved.

**Figure 4:**
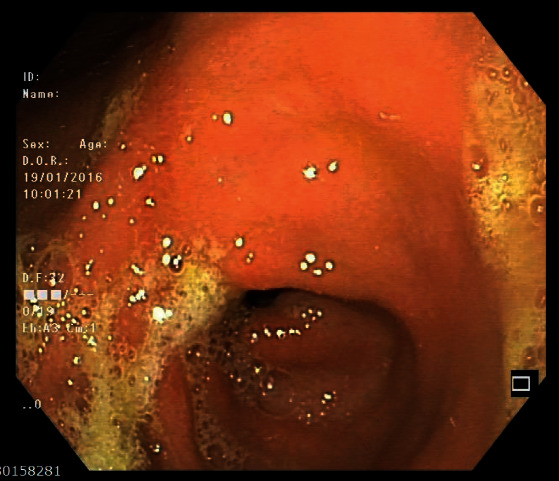
Follow-up endoscopy showing a residual scar.

Follow-up EGD, carried 50 days after the initiation of treatment, was only notable for a residual antral scar (Figure 4). Histology from obtained biopsies revealed gastric mucosa with mild chronic inflammation and normal duodenal mucosa. The patient completed the prescribed therapy for three months with no significant adverse or unanticipated events.

Follow-up 5 years later was carried out by examining his medical inpatient and outpatient records and by contacting him via phone. The patient was healthy, with no evidence of immunosuppressive state.

## 3. Discussion and Conclusions

While CMV infection in immunocompromised patients is associated with substantial morbidity and high mortality rate, immunocompetent individuals are usually asymptomatic [3].

The colitis [8] and esophagitis [9] are the most common GI manifestation areas of a CMV infection. It is uncommon in immunocompetent individuals, and most cases were reported in scenarios of critical illness or immunosuppression such as organ transplantation, long-term renal hemodialysis, HIV infection, hematological and solid malignancies, and immunosuppressive chemotherapies and radiotherapies, among others.

Gastroduodenal CMV infection is uncommon and rarely reported in immunocompetent hosts. A prospective study of 38 immunocompetent patients with gastroduodenal ulcerations failed to show CMV involvement, even as a superinfection within areas of previous peptic or NSAIDs-related mucosal injury [10].

We searched PubMed for case reports up to April 2021 using the following keywords: (cytomegalovirus) AND (gastric ulcer OR gastritis) AND (immunocompetent). Of the 25 search results, articles published in English on CMV gastric disease in immunocompetent patients older than 18 years were included. We found 7 cases eligible for inclusion, as summarized in Table 1. Similar to the patient we report, all patients were male; one was elderly and diabetic, and most of the remaining were in their late twenties and early thirties with no underlying disease.

CMV infection of the upper GI tract in immunocompetent hosts has been reported to cause multiple shallow gastric erosions and ulcers similar to *Helicobacter pylori*- and NSAIDs-related ulcers [15]. Giant obstructive ulcers, mimicking advanced gastric carcinoma or primary gastric lymphoma, were mostly reported in patients with AIDS [18, 19] and renal transplant recipients [20]. Differently, we describe a case of an immunocompetent patient presenting with an ulcer mimicking an advanced gastric malignancy.

The pathogenesis of CMV-related ulceration is ill-defined. Cytomegalic vasculitis, in which CMV infection induces ischemic injury in the involved endothelial cells, has been proposed as a possible mechanism of ulceration [21, 22].

Reported gastric CMV infections were mostly accompanied with systemic inflammatory symptoms, mainly low-grade fever and fatigue. Epigastric pain was also a predominant symptom, described in five of the seven included case reports [11, 13–15]. Our patient presented without fever or abdominal pain, and Vergara et al. describe a case where gastric ulcer was the sole manifestation of CMV infection in an otherwise asymptomatic healthy host [23]. Of note, febrile and symptomatic patients with acute infectious diseases rarely undergo invasive procedures. Thus, underdiagnosis may partly explain the few documented cases of CMV-related gastroduodenal ulcerations.

The diagnosis of gastric CMV ulcers is challenging due to their rarity and the absence of distinct morphological characteristics. If CMV infection is suspected, it is important to obtain biopsies from the ulcer base and examine the biopsy specimens for inclusion bodies using H&E staining and IHC. The latter method has a better sensitivity [13]. The different available diagnostic methods are not always in concordance. In our literature review, one case was reported where IHC was positive in the absence of inclusion bodies [13]. Quantitative PCR technique can help establish the diagnosis when histopathologic testing is inconclusive or negative [14]. Occasionally, repeated endoscopy and careful observation of deep biopsies are conducive to establish the diagnosis [16].

It is unclear whether CMV is the primary etiology of the gastric lesion or colonizes it post factum as an innocent bystander. Halme and colleagues showed that CMV-positive cells were found in duodenal biopsy specimens obtained from 19% of immunocompetent patients who underwent upper GI endoscopies for dyspeptic symptoms. No gastric CMV infection was found, and the histopathological findings in the CMV-positive mucosa were nonspecific and mild [24]. In the present case, we could not document any other potential causative agent and the inclusion bodies had disappeared in the specimens obtained from the healing lesion. Hence, from these findings and the active CMV infection documented by blood PCR, we concluded that CMV infection was the etiology of the gastric ulceration in our patient.

CMV-associated ulcers in the GI tracts of immunocompetent hosts often resolve rapidly on oral proton-pump inhibitors (PPIs). Antiviral therapy may be a reasonable second-line therapy in PPI refractory cases [11, 12]. The decision to use antivirals can be complex and challenging. Fyock and colleagues suggest an algorithm to determine whether or not to use antivirals based on several risk factors extrapolated from a meta-analysis of cases with CMV colitis in immunocompetent hosts [12, 25]. Male patients older than 55 years, pregnant women, or patients with comorbidities (diabetes, chronic kidney disease, or malignancies) may benefit from antiviral therapy [12]. However, our 66-year-old patient demonstrated complete endoscopic and histologic resolution within 2 months on PPIs monotherapy.

As in our case, the time to ulcer resolution was 10 weeks in most cases, similar to that of *Helicobacter pylori*-related ulcers [13].

In conclusion, in this case report, we describe a case of gastric CMV infection mimicking an advanced gastric tumor in an immunocompetent patient. Literature review yielded scarce reports on CMV-related gastric ulcers in patients with an intact immune system. Review of previous cases reveals the condition to be self-limiting and rapidly resolving. The diagnosis may be challenging and requires raised awareness of the clinician, due to the sometimes unrevealing nature of the standard biopsy and staining and the overall rarity of the condition. Whether its discovery should lead to a search for an immunocompromised state remains unknown and will require further data collection and a larger case series in the future.

## Figures and Tables

**Table 1:** Summary of published data from previous cases of gastric cytomegalovirus disease in immunocompetent patients.

Ref.	Age/gender	Comorbidities	Symptoms	Endoscopic findings	Histologic findings	Treatment	Follow-up endoscopy
Crespo et al. [11]	31/male	None	Epigastric pain, fever	Superficial erosions of the gastric antrum	Inclusion bodies and positive IHC staining	IV ganciclovir 5 mg/kg twice daily for 7 days	Full resolution, time of repeated endoscopy is not mentioned

Fyock et al. [12]	83/male	DM	Melena	Gastritis with multiple small antral, duodenal, and colonic ulcers	Inclusion bodies and positive IHC staining, culture, and PCR	21-day course of oral ganciclovir followed by IV administration	Partial healing of the duodenal ulcers after the antiviral therapy; full resolution 4 years later with the persistence of CMV

Ebisutani et al. [13]	33/male	None	Epigastric pain, low-grade fever, cough	Multiple gastric papules and a large irregularly shaped shallow ulcer	Positive IHC staining in the absence of inclusion bodies	Oral PPI (rabeprazole, 20 mg/day)	Small gastric ulcer on day 40; a residual ulcer scar on day 68

Matsui et al. [14]	29/male	N/A	Epigastric pain, fever	Multiple shallow gastric ulcers and mucosal erosions	Positive PCR for CMV-DNA in the absence of inclusion bodies	Oral PPI	Full resolution 2 months later

Himoto et al. [15]	31/male	None	Epigastric pain, fever	Multiple shallow gastric ulcers and mucosal edema	Inclusion bodies and positive IHC staining	Oral PPI (lansoprazole, 30 mg/day)	Full resolution 2 months later

Xiong et al. [16]	44/male	None	Epigastric pain, abdominal distention	Multiple erosions in the gastric antrum and thickening of the stomach wall	Inclusion bodies and positive IHC staining, negative PCR for CMV-DNA	IV ganciclovir for 3 months	Full resolution 3 months later

Yamamoto et al. [17]^†^	35/male	None	Infectious mononucleosis syndrome	Thickened and eroded mucosa throughout the stomach	Positive IHC staining in the absence of inclusion bodies	None	Improvement, time of repeated endoscopy is not mentioned

^†^CMV-EBV coinfection.

## Data Availability

All data generated or analyzed during this study are included within this article.

## Abbreviations

ACE-I: Angiotensin converting enzyme inhibitor
CBC: Complete blood count
CMV: Cytomegalovirus
CRP: C-reactive protein
EGD: Esophagogastroduodenoscopy
GI: Gastrointestinal
H&E: Hematoxylin and eosin
IHC: Immunohistochemistry
NSAIDs: Nonsteroidal anti-inflammatory drugs
PCR: Polymerase chain reaction
PPI: Proton-pump inhibitors.

## References

[1] Krech U. (1973). Complement-fixing antibodies against cytomegalovirus in different parts of the world. *Bulletin of the World Health Organization*.

[2] Bate S. L., Dollard S. C., Cannon M. J. (2010). Cytomegalovirus seroprevalence in the United States: the national health and nutrition examination surveys, 1988-2004. *Clinical Infectious Diseases*.

[3] Cohen J. I., Corey G. R. (1985). Cytomegalovirus infection in the normal host. *Medicine*.

[4] Horwitz C. A., Henle W., Henle G. (1986). Clinical and laboratory evaluation of cytomegalovirus-induced mononucleosis in previously healthy individuals. *Medicine*.

[5] Eddleston M., Peacock S., Juniper M., Warrell D. A. (1997). Severe cytomegalovirus infection in immunocompetent patients. *Clinical Infectious Diseases*.

[6] Limaye A. P. (2008). Cytomegalovirus reactivation in critically ill immunocompetent patients. *Journal of the American Medical Association*.

[7] Razonable R. R., Humar A. (2013). Cytomegalovirus in solid organ transplantation. *American Journal of Transplantation*.

[8] Siegal D. S., Hamid N., Cunha B. A. (2005). Cytomegalovirus colitis mimicking ischemic colitis in an immunocompetent host. *Heart & Lung: The Journal of Critical care*.

[9] Venkataramani A., Schlueter A. J., Spech T. J., Greenberg E. (1994). Cytomegalovirus esophagitis in an immunocompetent host. *Gastrointestinal Endoscopy*.

[10] Murray R. N., Parker A., Kadakia S. C., Ayala E., Martinez E. M. (1994). Cytomegalovirus in upper gastrointestinal ulcers. *Journal of Clinical Gastroenterology*.

[11] Crespo P., Dias N., Marques N., Saraiva da Cunha J. (2015). Gastritis as a manifestation of primary CMV infection in an immunocompetent host. *BMJ Case Reports*.

[12] Fyock C., Gaitanis M., Gao J., Resnick M., Shah S. (2014). Gastrointestinal CMV in an elderly, immunocompetent patient. *Rhode Island Medical Journal*.

[13] Ebisutani C., Kawamura A., Shibata N. (2012). Gastric ulcer associated with cytomegalovirus in an immunocompetent patient: method for diagnosis. *Case Reports in Gastroenterology*.

[14] Matsui Y., Sugino N., Kaneko H., Watanabe M., Miura Y., Tsudo M. (2010). CMV quantitative PCR in the diagnosis of CMV-associated AGML in an immunocompetent host. *Internal Medicine*.

[15] Himoto T., Goda F., Okuyama H. (2009). Cytomegalovirus-associated acute gastric mucosal lesion in an immunocompetent host. *Internal Medicine*.

[16] Xiong X., Liu F., Zhao W. (2019). Cytomegalovirus infective gastritis in an immunocompetent host misdiagnosed as malignancy on upper gastrointestinal endoscopy: a case report and review of literature. *Human Pathology*.

[17] Yamamoto S., Sakai Y. (2019). Acute gastritis caused by concurrent infection with Epstein-Barr virus and cytomegalovirus in an immunocompetent adult. *Clinical Journal of Gastroenterology*.

[18] Andreollo N. A. (2015). Obstructive gastric pseudotumor caused by cytomegalovirus in an AIDS patient: a case report and review of surgical treatment. *American Journal of Case Reports*.

[19] Pérez-Pereyra J., Morales D., Díaz R., Yoza M., Frisancho O. (2008). Giant gastric ulcer by cytomegalovirus in infection VIH/SIDA. *Revista de Gastroenterología del Perú*.

[20] Lin C.-J., Pan C.-F., Wu C.-J., Chen H.-H., Kao C.-R., Lee C.-C. (2008). A giant gastric ulcer mimicking carcinoma in a renal transplant recipient with CMV infection. *Southern Medical Journal*.

[21] Patra S., Samal S. C., Chacko A., Mathan V. I., Mathan M. M. (1999). Cytomegalovirus infection of the human gastrointestinal tract. *Journal of Gastroenterology and Hepatology*.

[22] Campbell D. A., Piercey J. R. A., Shnitka T. K., Goldsand G., Devine R. D. O., Weinstein W. M. (1977). Cytomegalovirus-associated gastric ulcer. *Gastroenterology*.

[23] Vergara M., Herrero J., de Torres I., Armengol J. R., Saperas E., Malagelada J. R. (1998). Gastric ulcers as the only manifestation of infection by cytomegalovirus in immunocompetent patients. *Journal of Gastroenterology and Hepatology*.

[24] Halme L., Arola J., Höckerstedt K., Lautenschlager I. (2008). Human herpesvirus 6 infection of the gastroduodenal mucosa. *Clinical Infectious Diseases*.

[25] Galiatsatos P., Shrier I., Lamoureux E., Szilagyi A. (2005). Meta-analysis of outcome of cytomegalovirus colitis in immunocompetent hosts. *Digestive Diseases and Sciences*.

